# Experimental Research and Development on the Natural Convection of Suspensions of Nanoparticles—A Comprehensive Review

**DOI:** 10.3390/nano10091855

**Published:** 2020-09-16

**Authors:** S. M. Sohel Murshed, Mohsen Sharifpur, Solomon Giwa, Josua P. Meyer

**Affiliations:** 1 Center for Innovation, Technology and Policy Research, Department of Mechanical Engineering, Instituto Superior Técnico, Universidade de Lisboa, 1049-001 Lisboa, Portugal; 2 Department of Mechanical and Aeronautical Engineering, Faculty of Engineering, University of Pretoria, Hatfield 0028, South Africa; josua.meyer@up.ac.za; 3 Institute of Research and Development, Duy Tan University, Da Nang 550000, Vietnam; 4 Department of Mechanical Engineering, Olabisi Onabanjo University, Ibogun 112104, Ogun State, Nigeria; sologiwa2002@yahoo.com

**Keywords:** nanofluids, stability, thermophysical properties, natural convection, thermal management systems

## Abstract

Suspensions of nanoparticles, widely known as nanofluids, are considered as advanced heat transfer media for thermal management and conversion systems. Research on their convective thermal transport is of paramount importance for their applications in such systems such as heat exchangers and solar collectors. This paper presents experimental research on the natural convection heat transfer performances of nanofluids in different geometries from thermal management and conversion perspectives. Experimental results and available experiment-derived correlations for the natural thermal convection of nanofluids are critically analyzed. Other features such as nanofluid preparation, stability evaluation and thermophysical properties of nanofluids that are important for this thermal transfer feature are also briefly reviewed and discussed. Additionally, techniques (active and passive) employed for enhancing the thermo-convection of nanofluids in different geometries are highlighted and discussed. Hybrid nanofluids are featured in this work as the newest class of nanofluids, with particular focuses on the thermophysical properties and natural convection heat transfer performance in enclosures. It is demonstrated that there has been a lack of accurate stability evaluation given the inconsistencies of available results on these properties and features of nanofluids. Although nanofluids exhibit enhanced thermophysical properties such as viscosity and thermal conductivity, convective heat transfer coefficients were observed to deteriorate in some cases when nanofluids were used, especially for nanoparticle concentrations of more than 0.1 vol.%. However, there are inconsistencies in the literature results, and the underlying mechanisms are also not yet well-understood despite their great importance for practical applications.

## 1. Introduction

**Figure 1:**
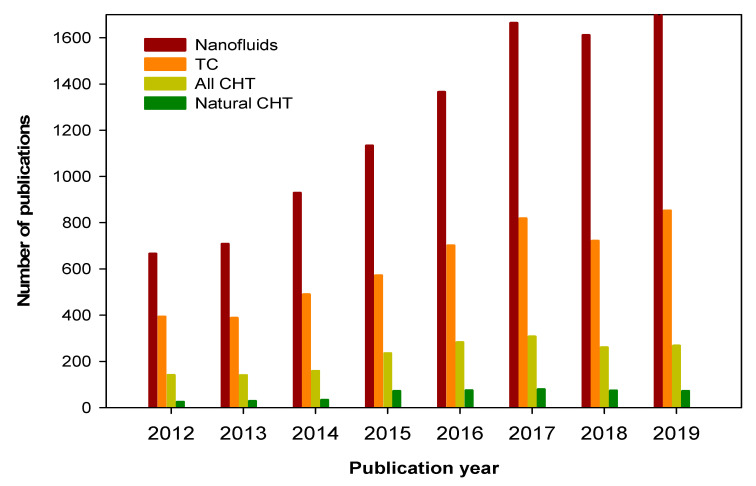
Publication records of NF, and their TCs and CHTs over the past several years (Web of Science).

Nanofluids (NF) which are considered as advanced cooling media, have received immense attention from researchers worldwide. Although the main application of this new class of fluids is in the thermal management and energy conversion systems, most of the research on nanofluids is on their thermal conductivity. Despite their huge importance and the potential research works related to their cooling applications and thermal energy conversion, such as applying them in solar thermal systems, research is still very limited. Figure 1 illustrates the global record of publication output regarding NF, including their thermal conductivity (TC), natural convection and convective heat transfer (CHT), plus applications of NF as obtained from the Web of Science platform (Clarivate) spanning 2012 to 2019. It is noted that the records of publications (Figure 1) include all types articles—experimental, theoretical, numerical and review studies. As can be seen from Figure 1 that research on NF has continued to grow, except for last year’s slight declination (for the first time), and TC is still dominating as the primary thermophysical property of NF being published. On the other hand, research on the CHT remained considerably behind the publications on the TC of NF with the ups and downs for the period considered. Furthermore, natural convection, which does not require the pumping power cost and is also a key cooling mode, has not received considerable attention from researchers (Figure 1). Additionally, natural convection studies of NF are significantly smaller compared with those of TC of NF and slightly less than those of CHT (forced convection). Thus, there is a clear need for more comprehensive works on the thermo-convection of NF under various thermal and concentration conditions in different geometries of cavities for their broad applications, particularly in thermal management and conversion.

Before employing NF in any application systems and characterizing their properties, NF must be engineered properly to ensure their long-term stability. Nanoparticles (NP) in a NF agglomerate and form sediments, which signify a poor degree of stability. This can negatively impact both the experimental measurements of the thermal properties and the convective behavior of NF in any geometry and flow conditions [1,2,3,4]. Basically, NF are formulated using single and two-step approaches [5,6,7]. The formal entails a single process of both synthesizing the NP and suspending the same in the base fluid (mainly at a volume concentration), whereas the latter involves a separate process of nanoparticles’ synthesis and their suspension into the base fluid. Since the stability of NP prepared by the two-step process is generally not satisfactory, it is important to undertake a proper dispersion process (e.g., sonication, surfactant addition, etc.) to ensure their improved stability.

Apart from the NF preparation and stability condition, the thermophysical properties, particularly TC and viscosity of NF, are very important for their convective heat transfer performance, and these properties of NF are considerably higher than those of their base fluids [8,9,10,11,12]. Numerous works have also been carried out on the CNT and flow under natural and forced (laminar, transition and turbulence) convection of NF in various geometries (cavities, ducts, macro- to micro-channels, etc.) [9,13,14,15,16,17]. Literature results showed even greater enhancement (compared with thermophysical properties) of these single-phase heat transfer features of NF [14,18]. In natural convection, studies are mostly performed using numerical simulations and only a handful conducted experiments, and the results are again not consistent and conclusive. Although NF’ research focuses have been expanded rapidly from properties characterization to energy harvesting, the main target of using NF in different engineering applications is to improve the cooling performances of conventional cooling media through increased convection heat transfer.

Tuckerman and Pease [19] invented the microchannel heat sink/system (MCHS) as an electronic cooling system, which was later investigated by Lee and Choi [20] for the enhanced cooling capability using novel NF. They experimentally demonstrated that the utilization of NF in an MCHS caused thermal cooling in addition to lower thermal resistance and higher power density compared with water [20]. The study of NF’s thermal transport behavior in an enclosure was pioneered by Putra et al. [6] who demonstrated experimentally that heat transfer was not enhanced but deteriorated when NF were used in a horizontal cylinder enclosure. However, no scientific test except visual inspection was conducted to check the stability of the prepared NF.

Another early experimental study on the thermo-convection of NF (TiO_2_/water) was conducted by Wen and Ding [7] in a mini-scale (several mm) enclosure. They measured the steady and transient heat transfer coefficients and reported a detraction of the convective heat transfer coefficient with a rise in particle concentration. Possible mechanisms (Brownian motion, slip, thermophoresis, electrophoresis, etc.) for such deterioration of heat transfer were attributed to convection brought about by the differences in concentration and temperature, particle/particle and particle/surface relations, changes of the dispersion properties, degree of stability and viscosity. The findings of these early studies have pioneered research in this area of NF. The stable TiO_2_/water NF was prepared by employing the pH control method while the zeta potential (ZP) technique was used to monitor the stability.

The engineering importance of thermal fluids in enclosures for cooling purposes has spurred the study into the heat transfer capabilities and behaviors of NF in cavities and channels of various shapes and sizes. Numerical studies have inundated the public domain regarding the deployment of NF as transport media in MCHS and cavities with the presence of very few experimental works. However, the experimental study is very vital for a deeper understanding of the physics and mechanisms of thermal cooling afforded using NF in addition to the contributions provided via computational and theoretical methods. The open literature remains uncertain concerning the heat transfer performance of NF in cavities under thermo-convection conditions while that of MCHS appears to be apparent.

Thus, this work took a specific interest in reviewing experimental studies based on the heat transfer capability of NF in diverse cavity geometries. We delved into the accuracy of measurements on the thermal properties and convective heat transport of NF in these enclosures. Stabilities of NF and experimental methods (intrusive and non-intrusive) and conditions were also reviewed and discussed. Additionally, correlations developed concerning the reviewed studies were provided. Furthermore, the stabilities, thermal properties and convection heat transfer performances of hybrid nanofluids (HNF) in cavities are presented and discussed in this paper.

## 2. Formulation and Stability of Mono-Particle and Hybrid Nanofluids

### 2.1. Nanofluids Formulation

The preparation of NF and HNF involves the suspension of NP of interest into specific base fluids. NF and HNF are formulated using the one-step and two-step approaches with the synthesis of NP for the latter carried out under different manufacturing techniques [21]. As NF or HNF are formulated by the dispersion of NP into conventional fluids (liquids), the stability of the resultant bi-phasic nano-based fluids is essential. The one-step approach involves the preparation of NF and HNF by the simultaneous synthesis of NP and dispersion in a base fluid. This approach offers the advantage of better stability and homogeneity of HNF and NF, and the elimination of laborious processes such as storing and drying compared to the one-step approach by reducing NP agglomeration [21,22]. Conversely, the industrial use of this method is not practicable except for fluids with low vapor pressure, and it is capital intensive [23].

The two-step approach of NF and HNF preparation has been mostly reported in the literature, especially for metallic oxide and carbon nanotube NP; this is due to the possibility of large-scale production of HNF and NF for industrial applications and economic efficiency. The demerit of this method has to do with sedimentation and agglomeration of the NP because of the Van der Waals force of attraction among particles [24]. Various manufacturing techniques used by numerous researchers under both the one-step and two-step approach of NF and HNF preparation have been well documented, and details can be found in the literature [25,26,27].

Preparation of NF and HNF using both methods requires agitation to achieve homogeneity and better stability of NF and HNF. The use of a homogenizer, a stirrer (magnetic), an ultra-sonicator and higher-shear devices to provide energy for even suspension of the NP into the base fluids is a must for both HNF and NF formulation [21,26]. Furthermore, the agitation time, intensity of agitation, volume/weight concentration or fraction, pH, temperature, surfactant types and quantities, combination (for HNF), sizes of NP and base fluid types are factors that are very important to the stabilities of the prepared HNF and NF [21,25,28,29,30,31].

### 2.2. Stability of Nanofluids

NF and HNF are adjudged to be electrically conducting fluids because electric charges are induced when NP are suspended in a base fluid. Thus, the formation of electrical double layers (EDLs) around the surface of the NP is strongly dependent on the volume concentration, surface and size charge of the NP, and ions’ concentrations in the base fluid [32]. The stability of NF and HNF is strongly linked to the thermal and convective properties of NF and HNF which consequently determine their application as heat transfer media [33]. The use of surfactants is to minimize the interfacial tension between NP and base fluid by increasing the EDL, thereby enhancing stability [25,28,34]. The choice of surfactants depends on the base fluid and NP types engaged in the formulation of NF [16,31,35,36]. It is worth stressing that the use of surfactants to improve the stability of NF and HNF is achievable at an optimum weight fraction above and below which instability is experienced [37,38,39]. The optimal weight fraction equivalent to the critical micelle concentration is very important to NF and HNF stability.

Experimental works on the convection heat transfer of NF and HNF involved the determination of both the thermal and convective properties, which required good stability of the NF and HNF. It can be simply put that the thermal properties (mainly viscosity and TC) of NF and HNF are dependent on the stability, while the CNT properties depend on the thermal properties, as has been duly reported in the literature [1,3,4,29,30,40]. To enhance the stability of NF, researchers have employed the use of diverse surfactants (e.g., sodium dodecyl sulphate and gum Arabic), pH and surface modifications (functionalization) [21,24,25,27,31,41,42,43]. Agitation periods (stirring and sonication) of a few minutes to a few hours have been reported in the literature [1,25,29,31,44]. It is worth noting that the optimum agitation time; surfactant quantities; temperature; combination of hybrid nanoparticles (HNP)—for HNP; and pH are key to the stability and thermophysical properties (TC, density and viscosity) of HNF and NF.

Experimental methods have been devised to check the stability of NF and HNF. These include sedimentation, centrifugation, spectral absorbency, viscosity, electron microscopy, ZP, poly-disparity index and light scattering [10,27,34,45,46,47,48]. In studies involving CHT in enclosures, the sedimentation, centrifugation, spectral absorbency, viscosity, density, ZP and poly-disparity index are mostly engaged to check the stability of NF and HNF [15,16,36,49,50,51]. To monitor the duration of stability of HNF and NF, the spectral absorbency, light scattering, density and viscosity methods are engaged while the on-the-spot stabilities of HNF and NF are inspected using other techniques. However, the visual method of stability inspection—which is not suitable to a large extent as it is not scientific—has been the most reported of the methods for NF and HNF stability monitoring. It is pertinent to report that there are some published works on the thermal properties and natural convection of NF that did not evaluate the stability of the studied NF [52,53,54,55].

In order to determine the dispersion/stability state of the colloidal suspensions, zeta potential is widely used. As a rule of thumb, nanofluids’ stability states are generally classified according to the zeta potential (in absolute value), as depicted in Table 1.

**Figure 2:**
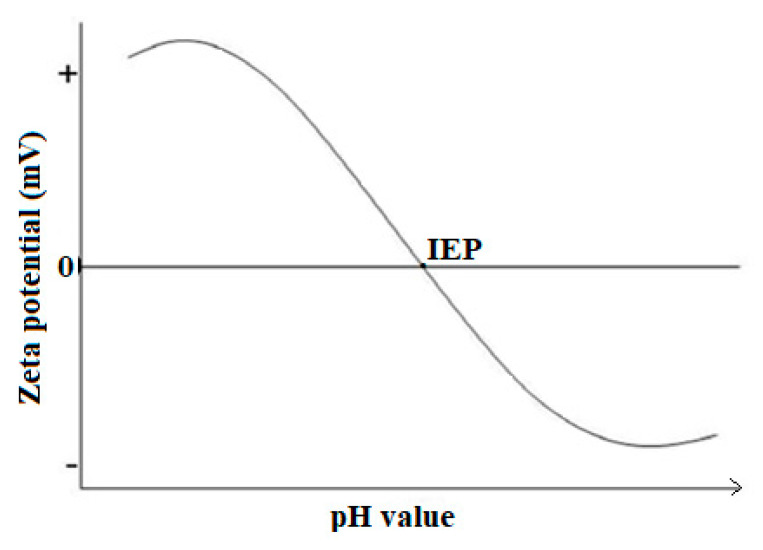
Typical zeta potential versus pH curve of suspension.

Researchers also confirmed that NF having ZP of above 30 mV (absolute value) showed relatively good stability character [15,52]. However, the ZP of suspensions can be significantly changed by using dispersion techniques such as ultrasonication and by changing the pH value. Figure 2 shows a typical ZP curve as a dependent of pH value could provide a better understanding of which value of pH can yield a large magnitude of ZP, and thus, better stability. It is noted that the iso-electric point (IEP) for NF may occur at much lower pH values than what is exemplified in Figure 2. A NF or HNF has better stability when its pH is distanced from the IEP. For example, good stability of Al_2_O_3_/distilled water (DW) NF was found at a pH value of above 8 which was the IEP [41].

## 3. Thermophysical Properties of Nanofluids and Hybrid-Nanofluids

Thermal properties of NF and HNF, namely, electrical conductivity, specific heat capacity, viscosity, TC, density, thermal expansion coefficient, etc., have been determined experimentally, with viscosity and TC playing prominent roles in thermo-convective heat transfer performances of NF and HNF inside various cavity geometries. Since extensive studies have been conducted on these two properties of NF and HNF, with limited works on other properties, a review of these properties as they relate to NF and HNF is briefly presented here; we also show summarized findings on thermophysical properties of HNF in Table 2.

### 3.1. Thermal Conductivity

Several researchers have contributed to the body of knowledge on the TC augmentation of NF and HNF. Early works showed TC improvement of up to 20% above the base fluids for NF when volume/mass fractions of less than 5% were studied [78,79,80,81,82]. Diverse mechanisms (clustering, thermal interfacial resistance, Brownian motion, interfacial Kapitsa resistance, percolation, aggregation, nano-layer, etc.) have been proposed for the uncharacteristic TC augmentation recorded for NF [83,84,85]. Masuda [81] pioneered the investigation of the TC of NF when 13 nm alumina NP were dispersed in water. An enhancement of 30% over the base fluid was recorded for 4.3 vol.%. Thereafter, several studies have been carried out using diverse NP (Cu, CuO, etc.) with different nano-sizes and various base fluids (water, ethylene glycol, etc.) to prepare NF, and they reported various degrees of enhancements under diverse ranges of temperatures and volume/mass fractions or concentrations. The literature revealed the enhancement of TC of NF in comparison with the respective base fluids as the temperature and volume/mass fraction or concentration increased [12,48,59,80,86,87,88,89,90,91,92,93,94,95]. The influence of magnetic field on the TC of NF has been investigated by a few researchers [91,92,93,96,97,98,99]. They reported that the TC was enhanced as magnetic field intensity was increased.

Advancement in research and the need to further improve the TC of NF has led to the idea of HNF as pioneered by Chokpar et al. [56] and Jana et al. [57]. By dispersing Al_2_Cu and Ag_2_Al HNP in water and Ethylene Glycol (EG), Chopkar et al. [56] revealed TC augmentation of 50% to 150% for *φ* = 0.2–1.5 vol.% in comparison with the respective base fluids. In contrast, Jana et al. [57] observed that dispersing NP (Cu, CNT and Au) and HNP (CNT–Cu and CNT–Au) in DIW yielded higher TC for NF than HNF. Several studies have been conducted on the TC of HNF since the pioneering works of Chokpar et al. [56] and Jana et al. [57]. These studies utilized several combinations of NP dispersed in diverse base fluids at different temperatures and volume/mass concentrations or fractions and reported TC enhancements with a rise in temperature and volume/mass concentrations or fractions [46,58,59,61,62,63,65,75,77,100,101,102]. It has been established and published that the TCs of HNF are numerically above those of NF [65,77,102]. The literature is very scarce in the open domain concerning the stimulus of the magnetic field on the TC. Shahsavar et al. [76] showed that exposing a magnetic HNF (Fe_3_O_4_-CNT/water) to increasing magnetic field intensity (0–480 mT) enhanced the TC in comparison to a no-magnetic field case. Hajiyan et al. [103] also showed a TC improvement of 16.9% for Fe_3_O_4_/glycerol NF at *φ* = 3.0%, 40 °C and the magnetic field intensity of 543 G.

Owing to the engineering, processing and energy system advantages, the measured data of the TC of NF and HNF were fitted using various techniques (regression, the response surface method, artificial neural networks, vector support machines, etc.) to propose models for the prediction of this property [47,62,65,100,103,104]. Despite a large number of predictive models being developed, so far no model is either widely accepted by the researchers or capable of predicting well the TCs of a wide range of NF [8,9,105]. The TC of NF was observed to be influenced by volume/mass fraction or concentration; NP type, shape and size; base fluid; temperature; etc.

**Figure 3:**
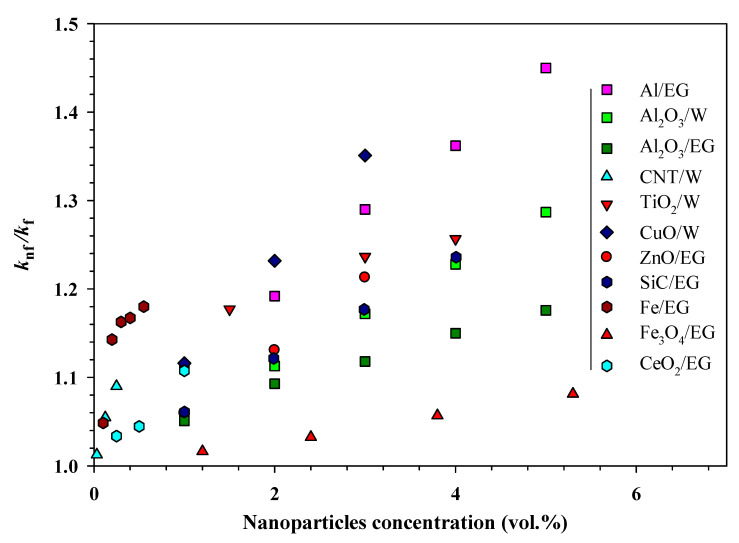
Enhanced thermal conductivities of various nanofluids as they relate to nanoparticle loading (abbreviation: CNT, carbon nanotubes; EG, ethylene glycol; W, water) (adapted from the authors’ earlier study [106]).

Despite being the most researched property of NF, TC results from various groups are not consistent but NF exhibit considerably higher TCs compared with the base fluids even when the concentration of the NP is very low. The enhanced TC further increased with the loading of NP (until some critical concentration) [8,9,12]. Some representative TC results (ratio of TCs of NF (*k*_nf_) and base fluids (*k*_f_)) are shown in Figure 3. However, the underlying mechanisms behind the observed increase in the measured TC are not yet fully understood.

### 3.2. Viscosity

The open literature showed that more studies had been conducted on the TCs of NF and HNF than the viscosities. The dispersion of NP and HNP into the base fluid leads to an enhancement of viscosity of the base fluid. Several factors have been reported to be responsible for the viscosity enhancement of NF compared with those of base fluids. These include volume/weight fraction, temperature, nano-size, interfacial nanolayer, etc., with a recent study revealing nano-confinement, nanoparticle–fluid interactions and viscous dissipation as other factors that have an effect on the viscosity of NF [107,108]. This viscosity enhancement affects the heat and flow characteristics of NF and HNF as the pumping power is increased. This appears to be one of the downsides of the applications of NF and HNF. The primary purpose of engaging NF and HNF as cooling media is because of the improved TC leading to the circulation of fewer coolant liquids with less pumping power. However, enhancing the viscosity of NF and HNF above a certain limit would take away from the benefit afforded by the augmentation of TC. Conclusively, the viscosity of NF and HNF has a substantial impact on the overall CHT performance.

Pak and Choi [109] pioneered the measurement of the viscosity of NF. They engaged DW-based Al_2_O_3_ and TiO_2_ NF (*φ* = 1–10 vol.%) and measured the viscosity at temperatures of 20 to 65 °C. At 10 vol.%, the viscosity of Al_2_O_3_/DW NF was enhanced by 200-fold while that of TiO_2_/DW NF was enhanced 3-fold, when compared with DW. Several studies have been conducted on the experimental determination of the viscosity of NF in relation to temperature and volume/mass concentration or fraction [10,29,42,89,95,110,111,112,113,114,115,116]. These works involved different types of NP dispersed in various base fluids and measured at different ranges of temperatures and concentrations or fractions. The results revealed that the viscosity of NF was greater than those of the corresponding base fluids and that as volume/mass fraction or concentration increased viscosity was enhanced. Additionally, an increase in temperature caused the augmentation of NF viscosity. Additionally, the viscosity of NF was found to be dependent on nanosize, shear rate and sonication time.

Again, progress in research has shown that the hybridization of NP could be utilized to manipulate the viscosity of NF. The viscosity of HNF has been investigated by numerous researchers as regards temperature, volume/mass concentration or fraction and mixing ratio of HNP [47,48,63,66,69,70,72,100,104,117,118]. Similarly to NF, the viscosity of HNF enhanced with volume/mass concentration or fraction and detracted with temperature. Few studies have been published regarding the influence of magnetic field on NF and HNF and viscosity was observed to enhance as the magnetic field intensity increased [76,97,99,103,119,120,121]. Experimental data of viscosity of NF and HNF at various temperatures and volume/mass concentrations or fractions have been fitted into models for the estimation of viscosity [47,72,100,104,110,116,118,120,122]. These models are useful in the design of thermal systems for engineering and processing applications.

**Figure 4:**
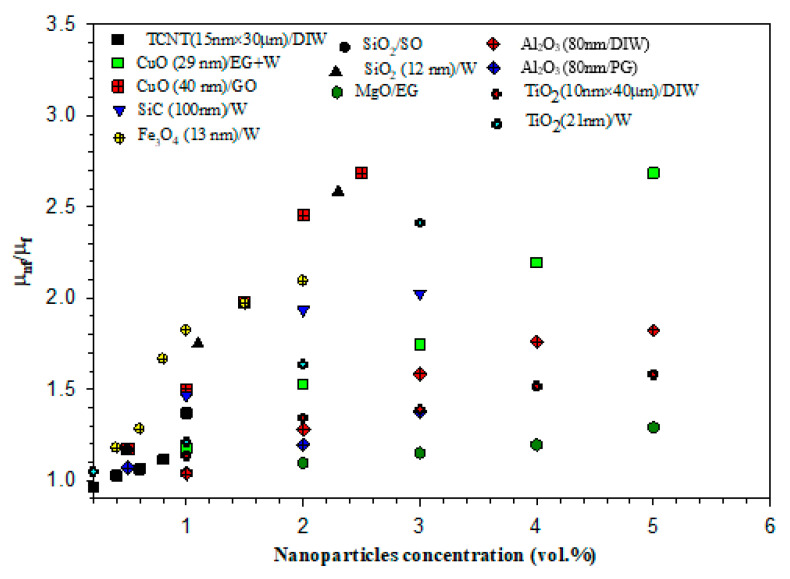
Enhanced viscosity of various nanofluids as regards nanoparticle loading (TCNT: treated carbon nanotubes, GO: gear oil, SO: silicone oil, PG: propylene glycol) (data adapted from an author’s previous study [11]).

The viscosities of NF are also crucial for their practical applications as cooling or heating media in flow systems. Some selective results of relative viscosity (ratio of the viscosity of NF (subscript, *nf*) and base fluids (subscript, *f*)) are shown in Figure 4, in which the data are from the author’s previous study [11]. In most cases, the viscosity of NF was found to decline with increasing temperature.

### 3.3. Other Properties

As earlier stated, TC and viscosity of NF and HNF are prominent properties that considerably impact the CNT and flow performance. Concerning the natural convection of NF and HNF in cavities of diverse geometries, the viscosity and TC of NF seem to be the most influential properties outside specific heat capacity, density and thermal expansion coefficient, engaged in the reduction of the obtained experimental data. Subject to this, other measured properties of NF and HNF are not known of in as much detail as those of TC and viscosity.

Density relates directly to the Nusselt number (*Nu*), Reynolds number (*Re*), pressure drop and friction factor of NF in CNT. Experimental measurements of the densities of NF and HNF have been carried out using different base fluids and NP or HNP at various temperatures and volume/mass concentrations or fractions [22,33,47,73,117,123,124,125,126,127,128]. The literature showed that the density of NF and HNF enhanced with the dispersion of NP or HNP in the different base fluids and detracted with a rise in temperature. This is because the NP and HNP are higher in density than the base fluids. Models for predicting the densities of NF and HNF have also been formulated [47,73,126,128].

The specific heat capacity of NF/HNF is important for analyzing the energy and exergy performances. With limited studies on the measurement of this property, two categories of base fluids (low and high temperature) are used for the dispersion of different NP and HNP at different temperatures and volume/mass concentrations or fractions. Generally, the authors demonstrated that the specific heat capacity of NF and HNF was either improved [64,129] or diminished [22,33,47,124,129] with an increase in volume/mass concentrations or fractions. These observations were due to the specific heat capacity values of NP and the nanolayer effect. Similarly, an increase in temperature was found to either enhance specific heat capacity or detract it [22,33,64]. Models for the prediction of specific heat capacity of NF and HNF were formulated by authors using the experimental data of specific heat capacity [22,47,124,130].

The electrical conductivity of NF/HNF is strongly connected to their stability [71,131]. Subject to the pioneering work of Maxwell in 1873 on the spherical particle suspensions of micrometer and millimeter sizes at low volume concentrations [125], further studies have been performed on the electrical conductivities of NF and HNF. With the preparation of different NF and HNF from various NP and base fluids, open literature on this subject showed that the electrical conductivity of NF/HNF was either augmented with temperature rise and volume/mass concentration or fraction [42,71,74,132,133] or independent of temperature [22,131], or detracted with an increase in volume/mass concentration or fraction [67,94].

Other measured properties of NF are flashpoint [51], contact angle [134], volumetric heat capacity [115], breakdown voltage [51], surface tension [135], extinction coefficient and transmittance [33] and shear stress [135], while those of HNF are flashpoint [68] and surface tension [47].

## 4. Convective Heat Transfer Performance of Nanofluids

Findings from available experimental studies on the convective heat transfer of NF in diverse geometries and under different flow regimes and thermal conditions have been critically analyzed. It was observed that most of the experimental studies employed square cavities. Thus, based on the geometry of each system available, findings and their discussions are grouped into four subsections: square, rectangular, cylindrical and other geometries. The influences of hybrid NF, aspect ratio (AR), bio-based NF, cavity inclination (*θ*), magnetic field intensity, porous media and magnetic field orientation on convective heat transfer and flow behavior in the different geometries of cavities are discussed herein. In addition, a detailed summary of natural convection heat transfer of nanofluids in various cavity geometries is presented in Table 3.

### 4.1. Convection in Square Cavities

Various configurations of cavities investigated for the natural convection heat transfer of different types of NF and HNF are presented in Table 4. The convective heat transfer behavior of ZnO/DIW-EG (*φ* = 5.25 wt.%) NF enclosed in a square-shaped enclosure was tested by Li et al. [144]. The results showed that the heat transfer capability of the NF was deteriorated in relation to the base fluid as the EG content was increased. In a vertical square cavity, Hu et al. [141] utilized DIW-based TiO_2_ (3.85–10.71 wt.%) NF to investigate the free convection heat transfer performance. They noticed the detraction of heat transfer for the NF when compared with DIW. Kouloulias et al. [52] tested the thermo-convection behavior in a square enclosure saturated with Al_2_O_3_/water (0.01–0.12 vol.%) NF. Their result also revealed the attenuation of heat transfer for the studied NF in relation to water. Nevertheless, the work of Garbadden et al. [16] reported enhancement of heat transfer when DIW-based MWCNT (0–1 vol.%) NF was engaged in a square cavity to investigate the thermo-convection performance. Maximum heat transfer enhancement of 45% at *φ* = 0.1 vol.% was reported. In addition, the convective heat transfer of Al_2_O_3_/DW (0.1–4 vol.%) NF in a square geometry with three different square cavities was tested by Ho et al. [145]. They demonstrated that heat transfer was enhanced at lower *φ* (maximum at 0.1 vol.%) for all the cavities and increased with cavity size. In comparison with DW, maximum heat transfer coefficient enhancement of 18% was achieved with the largest cavity. A correlation was developed from the experimental data for *Nu* estimation (see Table 5). On investigating the natural convection heat transfer behaviors of graphene, MWCNT and Al_2_O_3_/DW (0.1–0.5 vol.%) NF in a square cavity, Joshi and Pattamatta [142] also reported heat transfer augmentation of the NF relative to DW, which agreed with the works of Garbadden et al. [16] and Ho et al. [145].

The current trend of investigations on NF has established that the thermal and convective properties of NF can be improved through the utilization of HNF and an external magnetic field [48,55,76,150]. Thus, HNF as a passive method and a magnetic field as an active technique have been studied for possible augmentation of convective heat transfer of nano-engineered fluids in different square geometries. The influences of a uniform magnetic field on the thermo-convection characteristics in a square enclosure filled with a magnetic NF (Mg-Zn ferrite/kerosene) were examined by Yamaguchi et al. [154]. The imposition of the magnetic field was observed to increase thermal transport in the cavity, which was further enhanced with increased magnetic field intensity. By using an identical experimental set-up as Yamaguchi et al. [154] and mounting heat-producing objects within the cavity, Yamaguchi et al. [146] examined the natural convection thermal transport performance. An increase in the size of the heat-producing objects was observed to slightly decrease heat transfer. By engaging a non-magnetic NF (Ag/water) in a cubic enclosure and stimulated by a variable magnetic field, the influence of ΔT and the magnetic field on the thermo-convection performance was experimentally investigated [55]. The result demonstrated that the enhancement of *Nu* depended on ΔT and magnetic field strength. This finding was found to be contrary to that reported by Dixit and Pattamatta [143], as they revealed the attenuation of heat transfer on the exposure of non-magnetic NF (graphene/DW, SiO_2_/DW, MWCNT/DW, and Cu/DW; 0.057–2 vol.%) contained in a square enclosure of magnetic fields. However, without a magnetic field, they reported augmentation of heat transfer for DW-based graphene and MWCNT NF at 0.1 vol.%.

Recently, Dixit and Pattamatta [159] measured the impact of magnetic field orientation on the convective heat transfer performance in a square cavity containing DI-based Fe_3_O_4_ (0.05 vol.% and 0.2 vol.%) and Fe (0.2 vol.%) NF. Two cases of heating of the cavity and three cases of uniform magnetic field (0.3 T) exposure on the cavity were considered. The opposite vertical sides of the cavity were heated with the exposure of the magnetic field on the hot side and not-heated walls, while the magnetic field was only imposed on the hot face of the cavity when the top and bottom walls were differentially heated. They reported heat transfer attenuation upon exposing the heated and non-heated vertical walls to the magnetic field for both types of NF. However, heat transfer was enhanced by 11% and 28% upon exposing the heated bottom surface to the magnetic field for 0.05 vol.% and 0.2 vol.%, respectively. The orientations of the magnetic field and heated surface were observed to be accountable for the attenuation and enhancement of heat transfer for both types of NF. The provision of another path for the conduction of heat from the hot side to the cold side in the magnetic field direction due to chain formation was reported to be responsible for the thermal transport augmentation.

**Figure 5:**
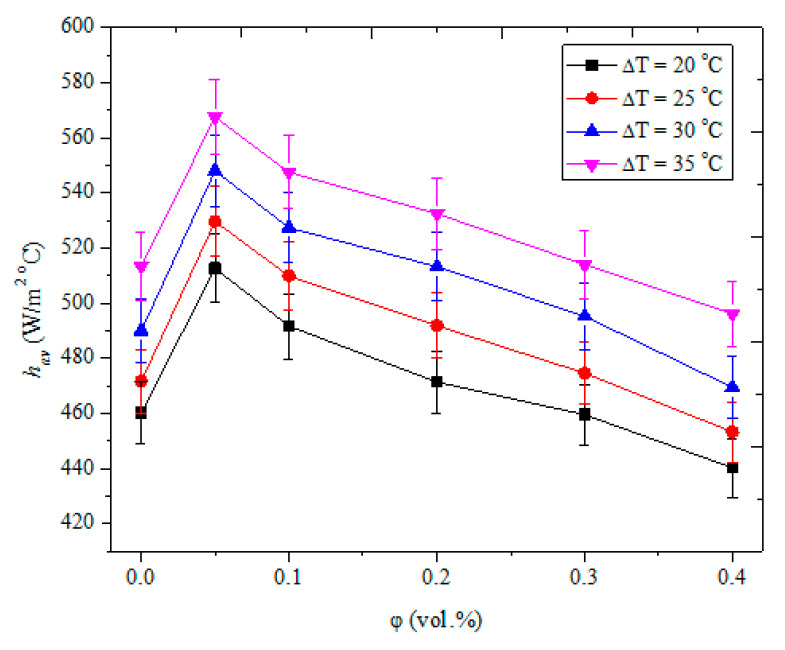
Average heat transfer coefficient of Fe_2_O_3_-MWCNT (80:20)/DIW NF in a rectangular cavity (data adapted from the authors’ previous study [37]).

The thermo-convection characteristics of HNF in an enclosure were pioneered by Giwa et al. [150]. They studied the thermal transport performance in a square enclosure containing DIW-based Al_2_O_3_-MWCNT (90:10 and 95:5) NF with *φ* = 0.1 vol.%. Their findings demonstrated that heat transfer of the HNF was augmented relative to DIW and DIW-based Al_2_O_3_ NF [15]. With the use of Al_2_O_3_-MWCNT (90:10)/DIW NF, the coefficient of heat transfer and *Nu* were augmented by 9.8% and 19.4%, respectively. Additionally, Giwa et al. [157] worked on the thermo-convection behavior in a square enclosure filled with DIW-based HNF (Al_2_O_3_-MWCNT). They experimented with DIW-based Al_2_O_3_:MWCNT (80:20–20:80) NF for 0.1 vol.% at *Ra* of 1.65 **×** 10^8^–3.80 × 10^8^. They found that the HNF with 60:40 mix ratio of Al_2_O_3_:MWCNT NP had the highest values for *Nu_av_* (16.2% increase) and *h_av_* (20.5% increase). They developed a correlation for predicting *Nu_av_* from experimental data. Figure 5 shows the effect of *φ* on the average heat transfer coefficient of DIW-based HNF (Fe_2_O_3_-MWCNT (80:20). Results of this HNF clearly demonstrate that the heat transfer coefficient increases considerably with increasing temperature difference. However, concentrations of both nanoparticles show interesting trends, where the smallest concentration (0.05 vol.%) yields the highest heat transfer coefficient which decreases with increasing concentration.

### 4.2. Convection in Rectangular Cavities

Ilyas et al. [136] studied the thermo-convection inside a vertical rectangular cavity (AR = 4) filled with thermal oil-based MWCNT (0–1.0 wt.%) NF. They reported that *h_av_* and *Nu_av_* were attenuated with an increase in *φ*. Deterioration of 21.3% and 35.7% was observed for *h_av_* and *Nu_av_* respectively, relative to thermal oil at *φ* = 1.0 wt.%. It was stressed that the observed deterioration was due to high enhancement of viscosity by 62% for *φ* = 1.0 wt.% despite the high TC afforded by MWCNT. Contrary to the results highlighted above, Ilyas et al. [147] and some others showed that heat transfer of NF in rectangular cavities was enhanced with an increase in volume concentration. Using thermal oil-based Al_2_O_3_ (0–3 wt.%) NF in a rectangular geometry with AR = 4, Ilyas et al. [147] revealed that heat transfer was enhanced as *h* increased from 1594 to 3175 W/m^2^ in comparison with thermal oil. Ho et al. [138] and Amiri et al. [51] reported that heat transfer was enhanced as the volume concentration increased when the thermo-convection behaviors of Al_2_O_3_/water (1–4 vol.%) NF and MWCNT-hexylamine/transformer oil (0.001–0.005 wt.%) NF filled into rectangular cavities were investigated. A correlation was proposed to estimate *Nu* as a function of some variables. Nnanna et al. [54] experimented the thermo-convection behavior of DIW-based Al_2_O_3_ (0.2–8 vol.%) NF in a rectangular enclosure and observed an enhancement of heat transfer for 0.2–2 vol.%, after which it detracted (>2%). The highest augmentation of heat transfer was achieved with 0.2 vol.%.

Ghodsinezhad et al. [15] reported the highest increase (15%) in convective heat transfer coefficient (HTC) for DIW-based Al_2_O_3_ (0.05–0.6 vol.%) NF inside a rectangular enclosure with 0.1 vol.% in relation to DIW. In addition, Rao and Srivastava [137] employed a non-intrusive method (interferometer) of measurement to study the thermo-convection of Al_2_O_3_/DIW (1–4 vol.%) NF in a rectangular geometry. They revealed that the convective HTC of the NF was enhanced by 38% in comparison to DIW. Additionally, increasing the volume concentration was observed to enhance heat transfer. The considerable enhancement in heat transfer achieved with a small temperature difference of 2.3 °C can be connected to the use of a non-intrusive temperature measurement technique in the cavity. It should be noted that this result is hard to come by using the intrusive method of temperature measurement via thermocouples. Additionally, the use of interferometric measurement by these authors led to the provision of conceivable explanations for the heat transfer augmentations accomplished by engaged Al_2_O_3_/DIW NF. Furthermore, they showed that three plausible mechanisms existed within the cavity and were accountable for the overall enhancement of the heat transfer rates recorded in the study. These were dominant convection structures, thermal boundary layer disruption phenomenon and TC enhancement.

Sharifpur et al. [17] tested the thermo-convection behavior of DIW-based TiO_2_ (0.05–0.8 vol.%) NF in a rectangular cavity and noticed that heat transfer was enhanced for 0.05–0.2 vol.%, and after that, it decreased. A maximum enhancement of 8.2% was recorded for 0.05 vol.%. Rao and Babu [156] examined the effects of heat inputs (30–50 W) and *φ* (0.05–0.6 vol.%) on the thermo-convection thermal transport behavior in a cylindrical cavity (AR = 0.0508) containing Al_2_O_3_/W NF. In comparison with water, their results exhibited enhancement of heat transfer for ≤0.1 vol.% and attenuation for >0.1 vol.%. The maximum *Nu* augmentation of 13.8% was recorded as *h* harmoniously increased from 382 W/m^2^–435 W/m^2^ for 0.1 vol.% and heat input of 50 W. A correlation was formulated to estimate *Nu* based on *Ra* and *φ*.

Some authors also studied the influences of AR and inclination angle (*θ*) as active techniques for the enhancement of thermo-convection performance of NF in rectangular cavities. Choudhary and Subudhi [139] studied the thermo-convection of Al_2_O_3_/DW (0.01 and 0.1 vol.%) NF in a rectangular cavity with varying AR (0.3 to 2.5). They revealed that *Nu* enhancement was dependent on *φ*, thermal boundary layer, AR and *Ra*. For both samples of NF, *Nu* was observed to be enhanced in comparison with DW. The highest enhancements of 29.5% (at AR = 0.5 and *Ra* = 7.89 × 10^8^) and 14.2% (at AR = 0.3 and *Ra* = 1.86 × 10^8^) were recorded for *φ* = 0.01 vol.% and 0.1 vol.%, respectively. Two correlations were formulated for the prediction of *Nu* and the thermal boundary layer of each NF (Table 5). Solomon et al. [49] examined the convective heat transfer behavior of DIW-based Al_2_O_3_ (0.1–0.6 vol.%) NF in an enclosure with varying AR (1, 2 and 4). They observed that heat transfer was sensitive to AR, *Ra* and *φ*. The cavity with AR = 1 (for 0.1 vol.%) had the highest augmentation of heat transfer. Qi et al. [140] also examined the effects of AR (0.25, 0.5 and 1) and *θ* (−45°–90°) on the thermo-convection of TiO_2_–water NF in a rectangular enclosure. They observed that the *Nu* was enhanced with power input and *φ*. Peak heat transfer was observed with the enclosure having AR = 1 and *θ* = 0°. In recent work, Torki and Etesami [40] examined the impacts of *φ* (0.01–1.0 vol.%), ΔT (2.3–30.9 °C) and *θ* (0°–120°) on the thermo-convection performance in a rectangular enclosure saturated with water-based SiO_2_ NF. The results showed maximum enhancement of *h* and *Nu* for *φ* = 0.01 vol.% at *θ* = 0°. Increasing *θ* (>0°) and *φ* (>0.01 vol.%) were observed to attenuate *h* and *Nu* while an increase in *Ra* and ΔT enhanced *h* and *Nu*. For the influence of *φ* on the natural convection performance of the NF in the cavity, increasing *θ* was observed to lead to deterioration.

Furthermore, the influence of porous media on the thermo-convection in a rectangular cavity containing Al_2_O_3_/EG (60%)-DIW (40%) (0.05–0.4 vol.%) NF was examined by Solomon et al. [149]. The result showed that the enhancement of heat transfer was a function of *φ* and porous media. By engaging the porous media and NF in the enclosure, heat transfer was enhanced by 10% with *φ* = 0.1 vol.% at ΔT = 50 °C, in comparison to DIW. Bio-based NF was investigated for the thermo-convection performance in a cavity. The pioneering work of Solomon et al. [148] in which a bio-based NF was engaged showed attenuation of heat transfer for all tested samples on investigating the hydrothermal behavior in a rectangular enclosure filled with mango bark/DIW NF (0.01–0.5 vol.%).

**Figure 6:**
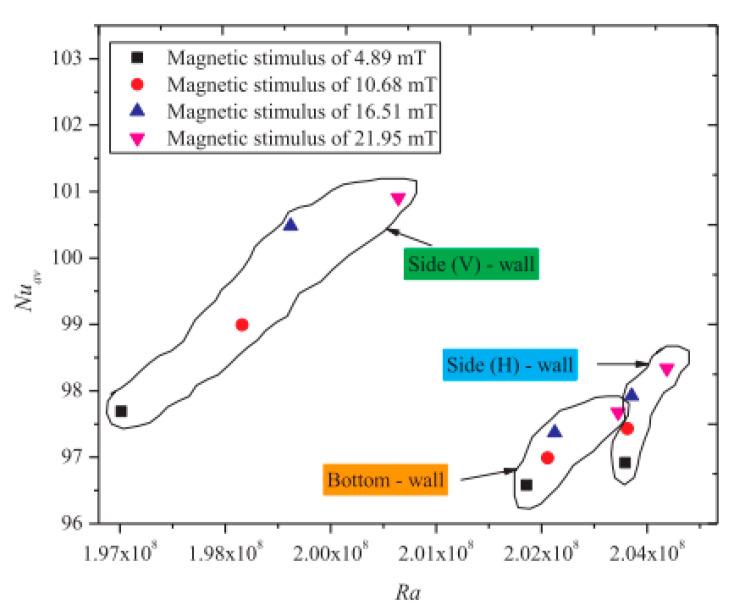
Influence of varying magnetic fields (imposed on different sides of a cavity) on heat transfer of Fe_2_O_3_-Al_2_O_3_ (75:25)/DIW NF in a rectangular enclosure (data adapted from author’s previous study [36]).

The active and passive utilization of a magnetic field and HNF, respectively, as potential techniques for the thermo-convection thermal transport augmentation in a rectangular geometry, were examined in the literature. The influences of *φ* and a magnetic field using three configurations of permanent magnets on the thermo-convection behavior in a rectangular cavity saturated with Fe_2_O_3_/DIW NF (0.05–0.3 vol.%) were investigated [35]. The results demonstrated that heat transfer enhancement pertaining to the NF depended on the magnets’ configuration, *φ* and magnetic field intensity. Maximum enhancement of heat transfer was achieved with *φ* = 0.1 vol.% when 700 G magnets were placed on the hot side (above and below) of the enclosure. On exposing the cavity to the magnetic field, *Nu* was improved by 2.81% (*φ* = 0.1 vol.%) in comparison with the case in which no magnetic field was applied. The combination of the magnetic field and HNF for convective thermal transport augmentation was performed by Giwa et al. [36]. They examined the impacts of a uniform magnetic field on the thermal transport behavior of Fe_2_O_3_-Al_2_O_3_ (75:25)/DIW NF in a rectangular enclosure. They worked using *Ra* range of 1.65 × 10^8^–3.80 × 10^8^, and observed heat transfer augmentations of 10.79% and 15.70% for cases without and with external uniform magnetic induction, respectively. Effects of magnetic field variation on the heat transfer capacity of DIW-based HNF (Fe_2_O_3_-Al_2_O_3_ (75:25) are presented in Figure 6, which shows the effect of magnetic field imposed on different sides of the cavity on the average *Nu* of this HNF.

### 4.3. Convection in Circular Cavities

Ni et al. [155] examined the turbulent thermo-convection of Al_2_O_3_/water NF in the classical Rayleigh–Bénard system with a cylindrical convection cell. They found a transition at *Ra_c_* (2.5 × 10^9^). When *Ra* > *Ra_c_*, almost no changes in *Nu* of NF (compared with water) were observed, but while at *Ra* < *Ra_c_*, the *Nu* of NF was found to be lower than that of water and the reduction in the trend was larger with decreasing *Ra*. They suggested that the significant decrease in the *Nu* of NF relative to water was due to the mass diffusion of NP. Ali et al. [5] examined the thermo-convection performance of aqueous Al_2_O_3_ NF with different volume concentrations (0.21–0.75 vol.%) inside vertical circular enclosures having AR = 0.0635 and 0.127 and heating below the cavities. They demonstrated that heat transfer was enhanced up to *φ* ≤ 0.51 vol.%, and the coefficient of heat transfer was also enhanced by AR. The heat transfer coefficients were noticed to be enhanced by 40% (when *φ* = 0.21 vol.% and AR = 0.0635) and 8% (with AR = 0.127 and *φ* = 0.51 vol.%), respectively. A correlation for the average *Nu* was proposed from their experimental results. On heating the same cavity engaged in the work of Ali et al. [5] on the top, Ali et al. [152] examined thermo-convection in a cylinder (having AR of 0.0635 and 0.127) saturated with DW-based Al_2_O_3_ (0.21–0.75 vol.%) NF. They demonstrated the attenuation of heat transfer of NF in the enclosure, which was related to AR and *φ* in relation to DW.

Putra et al. [6] examined the thermo-convection in a horizontal cylinder (with AR of 0.5–1.5) containing DW-based Al_2_O_3_ and CuO (*φ* = 1–4 vol.%) NF. The result showed that heat transfer was detracted for the NF samples. The attenuation observed was found to depend on AR, *φ* and nanoparticle density. Mahrood et al. [50] engaged carboxymethyl cellulose-based Al_2_O_3_ and TiO_2_ NF in a cylinder (having AR = 0.5–1.5) to study the convective heat transfer behavior. They reported the highest heat transfer for *φ* = 0.1 vol.% (TiO_2_ NF) and 0.2 vol.% (Al_2_O_3_ NF), with TiO_2_ NF appearing to be a better thermal fluid. By increasing AR, heat transfer was found to be enhanced for both types of NF.

A combination of two active techniques (*θ* and AR) for the improvement of heat transfer of NF in cavities was also examined. To study the impacts of heat fluxes (500–1500 W/m^2^), AR (0.5–1.5) and *θ* (30°–90°) on the thermo-convection performance in a cylindrical cavity heated from below, Moradi et al. [153] engaged DIW-based Al_2_O_3_ and TiO_2_ (*φ* = 0.1–1.5 vol.%) NF. An attenuation of the heat transfer for TiO_2_/DIW NF was observed, whereas heat transfer was augmented for Al_2_O_3_/DIW NF. Maximum heat transfer improvement was attained when *φ* = 0.2 vol.%, AR = 1 and *θ* = 30°.

### 4.4. Convection in Other Cavities

Most of the experimental studies on the thermo-convection transport of NF were performed with square, cylindrical and rectangular geometries, and only a couple of studies with other geometries can be found in the literature. The significance of two empirical models and an experiment-based model on the *Nu* and *h* ratio of SiO_2_/water (0.5–2.0 vol.%) NF in a triangular cavity was studied by Mahian et al. [3]. They showed that *Nu* deteriorated as *φ* increased, which was independent of *Ra*. At any *Ra*, the *h* of NF was observed to be higher than that of water. Additionally, the highest heat transfer for the NF occurred at *φ* = 0.5 vol.%. Moreover, the authors stressed the use of measured thermophysical properties in thermo-convection studies against those of empirical models. Umar et al. [160] performed experiments on the thermo-convection of water-based ZrO_2_ NF in triangular and rectangular sub-channels and reported augmentation of HTC of 5–10% for *φ* = 0.05 vol.% in comparison to water. Based on fitting their experimental data, they also introduced two correlations for the calculation of *Nu* in those geometries as a function of *Ra* and the hydraulic diameter of the channel.

Cadena-de la Peña et al. [151] examined the thermo-convection of MO-based AIN and TiO_2_ (0.01–0.50 wt.%) NF contained in a vertical annular cylinder. The result revealed that for both NF, *Nu* was enhanced when *φ* = 0.10 wt.% and it deteriorated at higher *φ*. The *h*_av_ and *Nu*_av_ of TiO_2_/MO NF were noticed to be higher than those of OA treated AIN/MO NF and AIN/MO NF. Both *h*_av_ and *Nu*_av_ were found to augment with an increase in *Ra* and a reduction in AR. The highest enhancements of *h*_av_ and *Nu*_av_ for TiO_2_/MO NF were noticed with *φ* = 0.10 vol.%, while those of AIN/MO NF occurred at *φ* = 0.10 vol.%, all at AR = 3.98. In addition, two correlations were formulated to estimate *Nu*.

**Figure 7:**
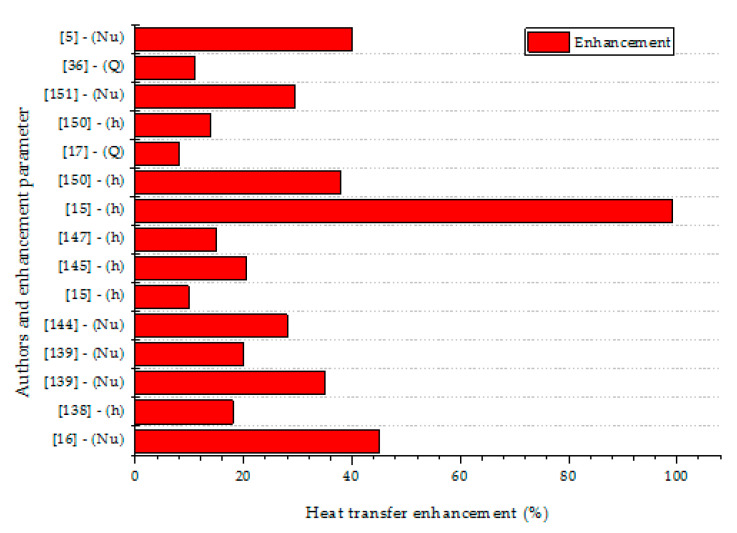
Heat transfer enhancements of different NF and HNF in diverse cavities.

The applicability of hemispherical enclosures for the management of convective heat transfer in electronic assembly and devices was investigated by Haddad et al. [158]. Haddad et al. [158] examined the impacts of *θ* (0°–180°) and *φ* (0.01–0.1 vol.%) on the thermo-convection thermal transport performance of water-based ZnO NF filling a hemispherical-shaped enclosure (with AR = 1.79) containing a cube-shaped object. The hemispherical dome was heated while the base was thermally insulated, and the object mounted inside the cavity was maintained at a cold temperature. They showed that the *Nu* was slightly augmented as *φ* increased. It was also observed that as *θ* increased, no noticeable enhancement of *Nu* was found. They also proposed a correlation to estimate *Nu* as a function of *Ra*, *Pr* and *θ* (see Table 5). Figure 7 provides the heat transfer enhancements of different NF and HNF in diverse cavities using different parameters.

The proposed correlations for predicting of *Nu* from *Ra*, volume/weight concentration, fraction, *Pr*, *Pr_r_*, *Κ_r_*, *β_r_*, etc., are provided in Table 5. It is to be noted that all these correlations were achieved by fitting the experimental data of the corresponding researcher(s). Subject to the afore-discussed studies, three characteristics of heat transfer have been reported in the open literature for thermo-convection of NF in cavities: deterioration and augmentation of heat transfer with volume/mass concentration, and the occurrence of optimum heat transfer with certain concentrations of NP (after which a decline was noticed) [6,16,137]. Nevertheless, no specific mechanisms behind such results were identified and discussed, except that reported by Rao and Srivastava [137].

## 5. Conclusions

In the context of this study, the following conclusions and remarks are highlighted:

The diversity in the preparation of NF and HNF in terms of the base fluid type, the size and type of NP, the agitation time and the method can seriously affect the stability, thermal properties and thermo-convection (heat transfer) of NF in cavities. There is outright non-uniformity in the manner NF preparation is reported in the literature, and hence, the preparations cannot be reproduced. Detailed and reproducible procedures for the preparation of NF are highly recommended in future studies. To reproduce NF or HNF, the sonication time, frequency, amplitude, stirring duration and weight fraction of surfactant (if used) are to be provided. Measurement of stability is suggested to be conducted before, during and after the thermo-convective experiment (and any properties or performance measurement).

Literature results showed three different characteristics of natural thermo-convection of NF: deterioration of heat transfer, especially for *φ* > 0.1 vol.% (in most cases), and the occurrence of optimum heat transfer at a certain *φ* value, after which a decline was noticed. The sensitivities of convective heat transfer of NF in various cavity geometries to viscosity and other variables, such as natural convection of base fluid, poor stability, high *φ* values, AR values of AR (in some cases), increase in *θ* and orientation of the magnetic field, have been attributed to the deterioration reported outside the slip mechanisms, Brownian motion, thermophoresis, etc. However, there is a clear scarcity of studies identifying the underlying mechanisms behind such results. On the other hand, each NF may enhance heat transfer in an exact volume fraction for an exact case (like natural convection) depending on the stability, base fluid, thermal condition, cavity geometry, etc. Therefore, the experimental results of a few volume fractions cannot conclude in general that an NF is good or bad, and more investigations are needed to conclude on a NF’s heat transfer capability in a known cavity geometry.

It is again suggested that the thermophysical properties used for data reduction should be experimentally determined for the NF being studied and not from empirical or theoretical or experimental models obtained from previous studies. It is observed that for the same type of NF, the NP size, preparation and stability are different, which may cause a change in the thermophysical properties and subsequently affect the thermo-convection performance. Future studies are to focus on the effects of other NP (apart from Al_2_O_3_ and MWCNT), HNF, porous media, bio (green)-nanoparticles, *θ*, magnetic and electric field intensities and orientations, AR, micro-organisms and base fluids (ionic, green and others) on the thermo-convective heat transfer performance in various cavity geometries. A combination of some of these enhancement techniques (passive and active) on the convective heat transfer in cavities would herald a new dawn in this field of study, particularly for thermal management and conversion systems.

Furthermore, it was noticed that the intrusive nature of temperature measurement via thermocouples mounted in the cavity can affect the convective flow and thermal transport and must be investigated further. Finally, depending on the cavity geometry and thermal condition imposed, alterations of AR, *θ*, *φ*, porous media, HNF and the magnetic field have been found to yield augmentation or attenuation of heat transfer, except for the green NF. The use of baffled and diverse types, partitions and other CNT-enhancing techniques is missing in the public domain in relation to this study.

From the state-of-the-art of nanofluids, there is a clear need for more systematic research on the thermal convection of NF under various conditions and cavities for their applications in thermal management and energy conversion systems.

## Figures and Tables

**Table 1:** Zeta potentials and stabilities of nanofluids.

Average Zeta Potential (±mV)	Stability State
<25	Flocculation or coagulation (within short time)
25 to 40	Physically stable
40 to 60	Good stability
>60	Very good to excellent stability

**Table 2:** Thermophysical properties of hybrid nanofluids.

Researchers	HNP (Ratio)	Base Fluid	Properties	Temp. (C)	*φ* (vol.%)	Enhancement (%)	Surfactant
Chopkar et al. [56]	Al_2_Cu, Ag_2_Al (Al = 70%; Cu & Ag = 30%)	EG & DIW	*κ*	Room temp.	0.2–1.5	50–150%	Oleic acid
Jana et al. [57]	Au, CNT, Cu, CNT-Cu & CNT-Au (1.5–2.5)	DIW	*κ*	Room temp	0.3 & 0.5 (CNT) 1.4 (Au) & 0.05–0.3 (Cu)	74 (Cu)	Laurate salt
Baby and Ramaprabhu [58]	f-MWCNT-f-HEG (50:50 wt.%)	DIW & EG	*κ*	25–50	0.5–5.0	20 (DIW); 3 (EG)	-
Harandi et al. [59]	f-MWCNT-Fe_3_O_4_ (50:50)	EG	*κ*	25–50	0.1–2.3	30	-
Esfe et al. [60]	Ag-MgO (50:50)	DW	*κ* & *μ*	Room temp	0–2	-	CTAB
Mousavi et al. [47]	CuO-MgO-TiO_2_ (5 diff. ratios)	DW	*κ*, *μ*, *ρ*, *C*_p_ & surface tension	15–60	0.1–0.5	-	SDS
Abbasi et al. [61]	MWCNT-Al_2_O_3_ (1:1)	DIW	*κ*	Room temp	0.1	20.68	GA
Zadkhast et al. [62]	MWCNT-CuO (50:50)	DIW	*κ*	25–50	0.05–0.6	30.38	-
Wei et al. [63]	SiC-TiO_2_ (50:50 wt.%)	Diathermic oil	*κ* & *μ*	17–43	0.1–1.0	8.39 (*κ*)	Oleic acid
Akilu et al. [64]	SiO_2_-CuO/C (80:20 wt.%)	GL-EG (60:40 wt.%)	*C*_p_, *κ*, & *μ*	30–80	0.5–2.0	1.15X (*μ*); 21.1 (*c*_p_); 26.9 (*κ*)	-
Kakavandi and Akbari [65]	MWCNT-SiC (50:50 wt.%)	W-EG (50:50 vol.%)	*κ*	25–50	0.05–0.75	33	-
Sundar et al. [66]	GO-Co_3_O_4_ (67:33 wt.%)	EG & DW	*κ* & *μ*	20–60	0.05–0.2	EG (*μ* = 1.42-times & *κ* = 11.85) & DW (*μ* = 1.70-times & *κ* = 19.14)	-
Qing et al. [67]	SiO_2_-G	Naphthenic mineral oil	*κ*, *μ*, & *σ*	20–100	0.01–0.08 wt.%	*κ* (80 (HNF) & 29 (NF)); *μ* (29.7 (HNF) & 12.87 (NF); *σ* (557–97)	-
Kumar et al. [68]	Cu-Zn (50:50)	EO, VO, paraffin	*μ*, *κ* & FP	30	0.1–0.5	Cu-Zn/VO (best)	SDS
Alirezaie et al. [69]	MWCNT-MgO (10:90)	EO	*μ*	25–50	0.0625–1.0	-	GA
Esfe and Sarlak [70]	CuO-MWCNT (85:15)	EO	*μ*	5–55	0.05–1.0	43.52	-
Sundar et al. [71]	ND-Ni (84:16 wt.%)	DW & EG	*σ*	24–65	0.02–0.1	199.52–200.23 (EG); 1339.81–853.13 (*κ*)	-
Zawawi et al. [72]	Al_2_O_3_-SiO_2_; Al_2_O_3_-TiO_2_; TiO_2_-SiO_2_	PAG	*μ*	30–80	0.02–1.0	*μ* = 20.50 (Al_2_O_3_-TiO_2_/PAG); *κ* = 2.41 (Al_2_O_3_-SiO_2_) at 30 °C.	-
Askari et al. [73]	Fe_3_O_4_-G	DIW	*μ*, *κ*, & *ρ*	20–40	0.1–1.0	14–32	-
Naddaf et al. [74]	G-MWCNT (1:1)	Diesel oil	*σ* & *κ*	5–100	0.05–0.5 wt.%	-	Oleic acid and HA
Nabil et al. [75]	TiO_2_-SiO_2_ (50:50 vol.%)	DW-EG (60:40 vol.%)	*κ* & *μ*	30–80	0.5–3.0	22.8 (*κ*); 62.6 (*μ*)	-
Shahsavar et al. [76]	Fe_3_O_4_-CNT (1:2; 1:1; 2:1)	W	*κ* & *μ*	25–35	-	45.41 (no magnet); 152.95 (magnet)	TMAH (Fe_3_O_4_) & GA (CNT)
Aparna et al. [77]	Al_2_O_3_-Ag (50:50; 30:70; 70:30)	DW	*κ*	25–52	0.005–0.1	23.82	PVP

**Table 3:** Summary of natural thermo-convection of nanofluids in various cavity geometries.

Researchers	NF (*φ*)	Cavity Dimension	Rayleigh Number (*Ra*)	Measured Thermal Properties	Preparation Method (Stability Test)	Remark
Kouloulias et al. [52]	γ-Al_2_O_3_/DIW (0.01–0.12 vol.%)	Cubic with 1 × 10^−3^ m^3^.	2.5 × 10^9^–5.2 × 10^9^	-	2-step (-)	*Nu* and *h* deteriorate with *φ* increase at different ΔT conditions.
Ilyas et al. [136]	MWCNT/Thermal oil (0–1 mass%)	Vertical rectangular (12 × 4 × 3 cm) with AR = 4.	2.5 × 10^5^–2.7 × 10^6^	*μ*, C_p_, β and *κ*	2-step (-)	Deterioration of *h_av_* (21.3%) and *Nu_av_* (35.74%) as *φ* increases despite high TC.
Rao and Srivastava [137]	Al_2_O_3_/DIW (0.01–0.04 vol.%)	Rectangular (l = 60 mm, b = 25 mm, h = 20 mm)	5.0 × 10^4^–3.5 × 10^5^	-	2-step (visual)	Enhancements of *h_av_* (28.43–38.03) and *Nu_av_* (1.92–14.97) of NF in comparison to BF with increasing *φ* at different ΔT regimes.
Ho et al. [138]	Al_2_O_3_/W (1–4 vol.%)	Vertical rectangular (l = 60 mm, b = 25 mm, h = 25 mm)	5.78 × 10^5^–3.11 × 10^6^	-	(visual)	Enhancement of *Nu_av_* with *φ*. Sedimentation has more impact than Brownian motion and Ludwig-Soret effect.
Amiri et al. [51]	MWCNT- hexylamine/TO ((0.001 and 0.005 wt.%)	Cubic (203 × 100 × 221 mm^3^)	ND	*μ*, *ρ*, PP, *σ*, *C*_p_, voltage breakdown, FP, and *κ*	2-step (UV-vis, ZP and poly-disparity index)	Both *Nu* and *h* are enhanced with *φ*.
Choudhary and Subudhi [139]	Al_2_O_3_/DW (0.01 and 0.1 vol.%)	Rectangular (120 × 120 × 365 (h) mm^3^) with AR (0.3–2.5)	10^7^–10^12^	-	2-step (visual)	At low *φ*, heat transfer is enhanced but deteriorated at high *φ*. Heat transfer is related to AR, *Ra*, and *φ*.
Qi et al. [140]	TiO_2_-W (0.1, 0.3 and 0.5 wt.%)	Three rectangles with AR = 0.25, 0.5 and 1, and inclined at −45°, 0°, 45° and 90°)	ND	-	2-step (UV-Vis, visual)	*Nu* is augmented with increasing *φ* and Q. Highest heat transfer is achieved using the cavity with AR = 1 and at 0°.
Hu et al. [141]	TiO_2_/DIW (3.85, 7.41 and 10.71 wt.%)	Vertical square (180 × 80 × 80 mm^3^)	4.04 × 10^7^–21.07 × 10^7^	*μ* and *κ*	2-step (-)	Heat transfer of NF is deteriorated when compared with the base fluid.
Joshi and Pattamatta [142]	Al_2_O_3_/DW, MWCNT/DW and Graphene/DW (0.1, 0.3 and 0.5 vol.%)	Square (40 × 40 × 200)	7 × 10^5^–1 × 10^7^	*μ* and *κ*	1-step (G), 2-step (Al_2_O_3_ and MWCNT) (visual)	At *Ra* = 10^6^, DW-based MWCNT and Graphene NF enhance heat transfer for 0.1 and 0.3 vol.%, whereas at *Ra* = 10^7^, only MWCNT/DW and Al_2_O_3_/DW NF reveal the same at similar concentrations.
Dixit and Pattamatta [143]	SiO_2_/DW, MWCNT/DW, Graphene/DW, and Cu/DW (0.057, 1, and 2 vol.%)	Cubic (25 × 50 × 50 mm^3^) + magnetic field (0.13 T and 0.3 T)	1 × 10^6^–1 × 10^7^	*μ* and *κ*	2-step (ZP)	Heat transfer is augmented for all the graphene samples and MWCNT at 0.1 vol.%, without magnetic field. Generally, heat transfer in all the NF samples is deteriorated with magnetic field.
Li et al. [144]	ZnO/EG-DW (75:25, 85:15 and 95:5 vol) (5.25 wt.%)	Square (180 × 80 × 80 mm^3^)	5.25 × 10^7^–1.08 × 10^8^	*μ* and *κ*	2-step (PVP)	Under the experimental condition, heat transfer is deteriorated with an increase in EG content.
Nnanna [54]	Al_2_O_3_/DIW (0.2–7.9 vol.%)	Cuboid (35 mm × 40.32 mm × 215 mm)	0.3 × 10^7^–3.2 × 10^7^	*μ* and *κ*	1-step (visual)	Heat transfer is augmented at low concentration of NF (0.2–2 vol.%) but detracts at higher concentration.
Ho et al. [145]	Al_2_O_3_/DIW (0.1–4 vol.%)	Cuboid (25 × 25 × 60, 40 × 40 × 90, and 80 × 80 × 180)	6.21 × 10^5^–2.56 × 10^8^	*μ*, ρ, and *κ*	2-step (-)	Enhancement of heat transfer at lower concentrations (0.1 and 0.3 vol.%) is observed, which increases with cavity size.
Yamaguchi et al. [146]	Mg-Zn ferrite/kerosene (ND)	Cubic (7.5 mm each side) with a heat-generating object (brass and square)	Gr = 0–160; Gr_m_ = 1.22 × 10^3^–4.4 × 10^4^	*μ*, ρ, C_p_, β, M, and *κ*	2-step (-)	Exposure to the magnetic field enhanced heat transfer and irrespective of the size of the heat-generating objects.
Sharifpur et al. [17]	TiO_2_/DIW (0.05–0.8 vol.%)	Rectangular (96 × 103 × 120 mm^3^)	4.9 × 10^8^–1.47 × 10^9^	-	1-step (-)	Heat transfer is enhanced for 0.05–0.2 vol.% and thereafter decreased, with maximum of 8.2% attained with 0.05 vol.% at ΔT of 50 °C.
Solomon et al. [49]	Al_2_O_3_/DIW (0.1–0.6 vol.%)	Rectangular with AR = 1,2 and 4.	6.9 × 10^6^–4.0 × 10^8^	-	1-step (UV-Vis and viscosity)	Enhancement of heat transfer is observed to be related to AR, *Ra* and *φ*. Highest heat transfer occurs at 0.1, 0.2 and 0.3 vol.% for AR = 1,2, and 4, respectively.
Ghodsinezhad et al. [15]	Al_2_O_3_/DIW (0.05–0.6 vol.%)	Rectangular (96 × 120 × 102 mm^3^)	3.49 × 10^8^–1.05 × 10^9^	*μ*	1-step (ZP, UV-vis and visual)	Enhancement of *h* up till 0.1 vol.% is observed. At 0.1 vol.%, *h* is 15% augmented compared to base fluid
Garbadeen et al. [16]	MWCNT/DIW (0–1 vol.%)	Cuboid (96 × 96 × 105 mm^3^)	1 × 10^8^	*μ* and *κ*	2-step (viscosity and visual)	Optimum heat transfer occurred at 0.1 vol.% with 45% enhancement of *h* relative to the base fluid.
Ilyas et al. [147]	f-MWCNT/THO (0.5–3 wt.%)	Cuboid (12 × 4 × 3 cm^3^)	4.43 × 10^5^–2.59 × 10^6^	*μ*, ρ, C_p_, and *κ*	2-step (-)	The *h* is enhanced as volume concentration increased whereas *Nu* is attenuated.
Solomon et al. [148]	Mango bark/DIW NF (0.01–0.5 vol.%)	Cuboid (120 × 96 × 103 mm^3^)	0.2 x10^8^–6 × 10^8^	*μ* and *κ*	2-step (UV-vis and viscosity)	Deterioration of NF is observed with increase in volume concentration.
Roszko andFornalik-Wajs [55]	Ag/DW (0.1 vol.%)	Cubical with 0.032 m under magnetic field (10 T)	2.5 × 10^6^–2.2 × 10^7^	-	2-step (-)	*Nu* is dependent on the magnetic field and structure of the flow. The energy transfer is altered because of the magnetic field.
Solomon et al. [149]	Al_2_O_3_/EG (60%)-DIW (40%) (0.05–0.4 vok%)	Cuboid (120 × 96 × 103 mm^3^)	3 × 10^3^–1.3 × 10^4^ and 1.2 × 10^8^–4 × 10^8^	*μ* and *κ*	1-step (UV-vis, viscosity and visual)	Heat transfer is enhanced by 10% for the porous cavity at 0.1 vol.% and ΔT = 50 °C, compared to the base fluid.
Joubert et al. [35]	Fe_2_O_3_/DIW (0.05–0.3 vol.%)	Rectangle (99 × 96 × 120 mm^3^) under magnetic field intensity of 300 G and 700 G.	1.77 × 10^8^–4.26 × 10^8^	*μ*	2-step (visual and viscosity)	Without magnetic field, *Nu* is maximally enhanced by 5.63% for 0.1 vol.% NF while with magnetic field, an additional maximum augmentation of 2.81% is recorded.
Giwa et al. [150]	MWCNT-Al_2_O_3_ (95:5 and 90:10)/DIW (0.1 vol.%)	Square (96 × 96 × 105 mm^3^)	2.27 × 10^8^–4.7 × 10^8^	*μ* and *κ*	2-step (-)	The HNF enhance heat transfer better than both NF of Al_2_O_3_/DIW and base fluid.
Putra et al. [6]	Al_2_O_3_/DW and CuO/DW (1 and 4 vol.%)	Horizontal cylinder (inner diameter = 40 mm) at AR = 0.5 and 1.	1.6 × 10^7^–9.2 × 10^7^	*ρ, μ, κ*, and *γ*	2-step (visual)	For both NF, heat transfer deteriorates as AR and concentration increased but decreased with *Nu.*
Ali et al. [5]	Al_2_O_3_/W (0.21, 0.51 and 0.75 vol.%)	Two vertical cylinders (D = 0.2 m) with AR = 0.0635 and 0.127. Heated on the top wall.	3.0 × 10^5^–1.3 × 10^8^	*ρ, μ,* and *κ*	1-step (-)	The *Nu* and *h* of the NF are more deteriorated than the base fluid, which is related to volume concentration and AR.
Cadena-de la Peña et al. [151]	AIN and TiO_2_/mineral oil (0.01, 0.1 and 0.5 wt.%)	Annular and vertical (opened) with *AR* of 3.98 and 4.78.	1.4 × 10^9^–3.2 × 10^13^	*μ,* and *κ* (at 24 and 40 °C)	2-step (visual)	*Nu_av_* and *h_av_* are improved relative to the base fluid at certain conditions (low AR and φ, and high *Ra*). TiO_2_/mineral oil NF (*h_av_* = 2.63 -5.35% and *Nu_av_* = 3.45% maximum) performing better than the AIN/mineral oil NF (*h_av_* = 3.91% maximum)
Ali et al. [152]	Al_2_O_3_/W (0.21, 0.51 and 0.75 vol.%)	Two vertical cylinders (D = 0.2 m) with AR = 0.0635 and 0.127. Heated at the bottom.	3.0 × 10^5^–1.3 × 10^8^	*ρ, μ,* and *κ*	1-step (visual)	Compared to the base fluid, *h* is augmented for 0.21 vol.% and attenuated with concentration increase. HTC is AR dependent with higher *h* for lower AR.
Wen and Ding [7]	TiO_2_/DW (0.8, 1.5, and 2.5 wt.%)	Horizontal cylinder (240 mm diameter)	2.3 × 10^4^–1.4 × 10^5^	*μ* and *κ*	2-step (ZP and visual)	HTC attenuates with increase in NF concentration with maximum reduction of 30% recorded.
Mahian, et al. [3]	SiO_2_/W (0.5, 1.0, and 2.0 vol.%)	Square, inclined square (45°) and triangular	1.0 × 10^5^–1.0 × 10^6^	*ρ, μ,* and *κ*.	2-step (visual)	For all the cavities, the maximum HTC ratio is observed at Ra = 10^6^ and 0.5% concentration. High prediction accuracy of the HTC is noticed when the thermophysical properties of the NF are measured.
Mahrood et al. [50]	Al_2_O_3_ and TiO_2_/CMC (0.1 ≤ φ ≤ 1.5 vol.%)	Vertical cylinder with AR = 0.5, 1.0 and 1.5.	4.0 × 10^6^–3.0 × 10^7^	n.d.	2-step (-)	Heat transfer is enhanced below 0.5 and 1 vol.% with optimum values at 0.1 and 0.2 vol.%, for CMC-based TiO_2_ and Al_2_O_3_ NF, respectively. TiO_2_ NF is a better heat transfer medium than Al_2_O_3_ NF. Increasing *AR* is found to enhance heat transfer for both NF.
Moradi et al. [153]	Al_2_O_3_/DIW and TiO_2_/DIW (0.1 ≤ φ ≤ 1.5 vol.%)	Inclined (30°, 60° and 90°) vertical cylindrical (diameter = 80 mm and length = 250 mm) with AR (0.5, 1.0 and 1.5)	1.2 × 10^8^–3.7 × 10^8^	*Ρ*	2-step (visual)	Maximum enhancements of *Nu* (6.76% and 2.33% relative to DIW) occur at 0.2 vol.% and 0.1 vol.% for Al_2_O_3_/DIW and TiO_2_/DIW NF, respectively. *Nu* is noticed to augment with increase in AR.
Yamaguchi et al. [154]	Mg-Zn ferrite/alkyl-naphthalene	Cubic with a magnetic field.	Ra (3.0 × 10^3^–8.0 × 10^3^), Ra_m_ (1.0 × 10^8^–1.25 × 10^8^)	-	2-step (-)	Heat transfer is enhanced on exposure to magnetic field. An increase in the magnetic strength enhanced heat transfer further.
Ni et al. [155]	Al_2_O_3_/W (0.0108 vol.%)	Cylindrical (ID = 19.3 cm, h = 2.00 cm)	2.6 × 10^8^–7.7 × 10^8^	-	1-step (-)	Deterioration of *Nu*.
Babu and Rao [156]	Al_2_O_3_/DIW (0.05–0.6 vol.%)	Vertical cylinder (D = 12.7mm, l = 250mm)	2.7 × 10^9^–6.4 × 10^9^	-	2-step (UV-vis)	Improvement of heat transfer by 13.8% for 0.1 vol.%.
Torki and Etesami [40]	SiO_2_/DIW (0.01–1.0 vol.%)	Inclined rectangle (60 × 60 × 135 mm^3^)	1.0 × 10^7^–8.0 × 10^7^	-	2-step (-)	Maximum *h* and *Nu* at *φ* = 0.01 vol.% and *θ* = 0°. Attenuation are observed at >0.01 vol.% and >0°.
Giwa et al. [157]	Al_2_O_3_-MWCNT (80:20–20:80) (0.1 vol.%)	Square (96 × 96 × 105 mm^3^)	1.65 × 10^8^–3.8 × 10^8^	*μ* and *κ*	2-step (UV-vis)	Maximum enhancements of 16.2%, 19.4%, and 20.5% are reported for *Nu_av_*, *Q_av_*, and *h_av_*, respectively, for Al_2_O_3_-MWCNT/DIW NF with 60:40 percent weight of NP.
Haddad et al. [158]	ZnO/W (0.01–0.1 vol.%)	Inclined hemisphere with a cubical object.	5.21 × 10^7^–7.29 × 10^10^	*μ*, *κ*, Cp, β, *ρ*, and B.	2-step (-)	Heat transfer is slightly enhanced with an increase in *φ* while increasing *θ* does not affect it.
Giwa et al. [36]	Al_2_O_3_-Fe_2_O_3_/DIW (0.05–0.3 vol.%)	Rectangular (120.8 × 99.7 × 113.2 mm^3^) with a magnetic field of 48.9 G–219.5 G.	1.49 × 10^8^–3.04 × 10^8^	*μ*, *κ*, and B.	2-step (UV-vis)	Without magnetic induction, heat transfer is enhanced by 10.79% for 0.1 vol.% while in the presence of the magnetic induction heat transfer is further enhanced. Imposing the magnetic field vertically on the side wall of the cavity led to maximum heat transfer.
Dixit and Pattamatta [159]	Fe_3_O_4_/DI (0.05 and 0.2 vol.%) and Fe/DI (0.2 vol.%)	Cubic (25 mm each) with a magnetic field of 0.3 T.	4.23 × 10^5^–1.0 × 10^7^	*μ*, *κ*, and B.	2-step (ZP)	For both types of NF, deterioration is observed on exposing the vertical walls (heated and non-heated) to the magnetic field. Heat transfer is enhanced by 11.0% (0.05 vol) and 28% (0.2 vol) for Fe_3_O_4_/DI NF on exposing the heated bottom wall to the magnetic field.

**Table 4:** Different types of enclosures used in studying natural convection of nanofluids.

Enclosure	Configuration	Remark (References)
Rectangle	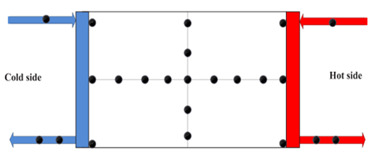	Natural convection of hybrid nanofluid (e.g., [36,40])
Square	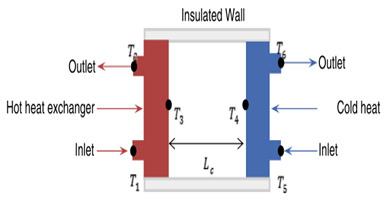	Natural convection of mono nanofluid (e.g., [16,137,143])
Cylinder	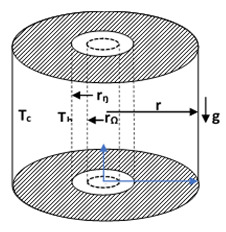	Natural convection of mono nanofluid (e.g., [151])
Hemisphere	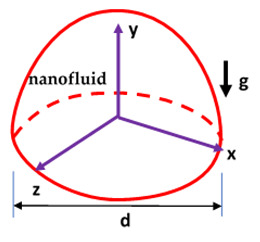	Natural convection of mono nanofluid with and without cavity inclination (e.g., [158])

**Table 5:** Proposed correlations for natural thermo-convection of nanofluids in different cavities.

Researchers	NF	Geometry	Correlation
Ali et al. [152]	Al_2_O_3_/W	Vertical cylinders	$Nu=\left(7.899-8.571\times 10^{-9}Ra^{*}\right)\times \left(1.0-15.283\varphi +387.681\varphi^{2}\right)AR^{0.5}$
Ho et al. [145]	Al_2_O_3_/W	Vertical squares	$Nu_{nf}=CRa_{nf}{}^{n}{\left(Pr_{nf,h}/Pr_{nf}\right)}^{m}{\left(\beta r_{nf,h}/\beta r_{nf}\right)}^{p}$
Cadena-de la Peña et al. [151]	AIN and TiO_2_/mineral oil	Opened vertical annular	$Nu=0.496Ra^{*}{}^{0.17}K^{\left(\frac{1.582}{k}+2.463\right)}AR^{-0.541}$ Maximum deviation = 1.6%
Ali et al. [5]	Al_2_O_3_/W	Vertical cylinders	$Nu=1.426{\left(Ra^{*}\right)}^{0.119}\left(1+44.097\varphi -6943.36\varphi^{2}\right)AR^{0.137}$
Ghodsinezhad et al. [15]	Al_2_O_3_/W	Square	$Nu=0.6091{\left(Ra\right)}^{0.235}{\left(\varphi \right)}^{0.00584}$ (for φ ≤ 0.1) $Nu=0.482{\left(Ra\right)}^{0.2356}{\left(\varphi \right)}^{-0.026}$ (for φ ≥ 0.1); R^2^ = 0.94
Ilyas et al. [147]	f-Al_2_O_3_/THO	Rectangular	$Nu=C{\left(Ra\right)}^{0.04}{\left(1-\varphi \right)}^{-0.015}$; 228 ≤ Pr ≤ 592; 0.97 ≤1− φ (wt. frac.) ≤ 1; $C=4.17{\left(\frac{Pr_{r}}{Pr_{r}-0.343}\right)}^{2.51}\left(\frac{Pr_{r}}{K_{r}{}^{3.76}\beta_{r}{}^{3.483}}\right)$
Nnanna et al. [54]	Al_2_O_3_/DIW	Rectangular	$Nu=16.4e^{-Ra\epsilon \varphi \left(e^{-m\varphi }\right)}$ ε = 4 × 10^−7^; m = 11; 10^5^ ≤ φ*Ra*e^−mφ^ ≤ 10^6^
Babu and Rao [156]	Al_2_O_3_/DIW	Vertical cylinder	$Nu=1.349\;{\left(Ra\right)}^{0.22}\;$(0 < *φ* < 0.10 vol.%); R^2^ = 0.995 $Nu=1.246\;{\left(Ra\right)}^{0.224}\;$(0.1 < *φ* < 0.60 vol.%); R^2^ = 0.997
Choudhary and Subudhi [139]	Al_2_O_3_/DW	Square	$Nu=0.1199Pr^{-1/2}{\left(Ra\right)}^{1/4}+2.17\times 10^{3}Pr^{-1/7}{\left(Ra\right)}^{3/7}$ (*φ* < 0.01 vol.%) $Nu=0.132Pr^{-1/2}{\left(Ra\right)}^{1/4}+1.66\times 10^{3}Pr^{-1/7}{\left(Ra\right)}^{3/7}$ (*φ* < 0.10 vol.%) ${\left(\frac{\delta_{th}}{H}\right)}_{NF}=122.55\;{\left(Ra\right)}^{-0.427}$; (*φ* < 0.01 vol.%) ${\left(\frac{\delta_{th}}{H}\right)}_{NF}=44.873\;{\left(Ra\right)}^{-0.3728}$; (*φ* < 0.10 vol.%)
Rao and Babu [156]	Al_2_O_3_/W	Cylinder	$Nu=1.349{\left(Ra\right)}^{0.22}$ (0 ≤φ≤0.1) $Nu=1.246{\left(Ra\right)}^{0.224}$ (0.1 ≤φ≤0.6)
Giwa et al. [36]	Fe_2_O_3_-Al_2_O_3_/DIW	Rectangular	$Nu=0.721{\left(Ra\right)}^{0.2429}\varphi^{-0.0613}$
Haddad et al. [158]	ZnO/W	Hemisphere	$Nu\left(\alpha \right)=\kappa \left(\alpha \right){\left(Ra\right)}^{b\left(a\right)}Pr^{m}$ $\kappa \left(\alpha \right)=6\times 10^{-6}\alpha^{2}-8\times 10^{-4}\alpha +0.4$ $b\left(\alpha \right)=-3\times 10^{-7}\alpha^{2}+2\times 10^{-6}\alpha +0.223$; m=−0.03 Valid for $5.21\times 10^{7}\leq Ra\leq 7.29\times 10^{10}$; 0°≤α≤180°; 3.95≤Pr≤6.07
Giwa et al. [157]	Al_2_O_3_-MWCNT/DIW	Square	$Nu=6.47\times 10^{-4}\;{\left(Ra\right)}^{0.5928}R^{0.00169}$
Umar et al. [160]	ZrO_2_/water	Triangular and rectangular	$Nu=16.22{\left(\frac{RaD_{h}}{x}\right)}^{0.0696}$ and $Nu=10.09{\left(\frac{RaD_{h}}{x}\right)}^{0.0702}$
